# Supplementary material for: Effects of maternal exercise on infant mesenchymal stem cell mitochondrial function, insulin action, and body composition in infancy
Source: Physiol Rep. 2024 Apr 29;12(9):e16028. doi: 10.14814/phy2.16028 (PMC11058002; doi:10.14814/phy2.16028)
Supplement: Supplementary file 1 — Table S1. Table S2. Figure S1. Figure S2. Figure S3. Figure S4. Figure S5. Figure S6. Figure S7. [file PHY2-12-e16028-s001.docx]

| **Supplementary Table 1. Western blot antibody information.** | | |
| --- | --- | --- |
| **Antibody** | **Manufacturer** | **Cat. No.** |
| Akt (Ser473) | Cell Signaling | 9271 |
| Akt (Thr308) | Cell Signaling | 4056 |
| Akt protein | Cell Signaling | 9272 |
| AMPK (Thr172) | Cell Signaling | 2531 |
| AMPK protein | Cell Signaling | 2532 |
| PGC1α | Abcam | ab106814 |
| SIRT1 | Cell Signaling | 2493 |
| Citrate Synthase | Abcam | ab96600 |
| Pyruvate Dehydrogenase | Cell Signaling | 2784 |
| Total OXPHOS | Abcam | ab9110411 |
| β-Actin | Cell Signaling | 4967 and 3700 |

| **Supplementary Table 2. Maternal and Infant Characteristics** | | | | |
| --- | --- | --- | --- | --- |
| **Maternal Characteristics** | **AE (10)** | **CE (9)** | **RE (11)** | **p-value** |
| Age (yrs) | 30.5±4.6 | 28.7±1.8 | 31.6±3.0 | 0.18 |
| VO2peak (ml/kg/min)* | 25.2±3.2 | 24.9±6.0 | 22.5±5.9 | 0.54 |
| Average MET*min/wk between 16-36 weeks of gestation | 551.4±96.8 | 597.8±83.31 | 572±110.7 | 0.60 |
| Total MET*min between 16-36 weeks of gestation | 11785±4413 | 13913±2932 | 12572±2473 | 0.4 |
| Pre-pregnancy BMI | 23.6±2.3 | 25.6±4.5 | 24.7±4.5 | 0.52 |
| 16-week BMI | 24.8±2.7 | 26.7±4.8 | 26.1±5.1 | 0.64 |
| 16-week Body Fat (%) | 35.1±3.2 | 33.5±4.5 | 33.2±4.6 | 0.58 |
| 16-week Total Cholesterol (mg/dl) | 184.6±27.1 | 159.4±20.2 | 184.9±37.6 | 0.13 |
| 16-week LDL (mg/dl) | 108.9±31.2 | 78.4±16.7 | 96.6±31.4 | 0.08 |
| 16-week HDL (mg/dl) | 52.3±19.1 | 62.7±7.9 | 69.1±15.4 | **0.05** |
| 16-week non-HDL (mg/dl) | 132.3±30.9 | 97.1±18.5 | 115.3±34.6 | 0.06 |
| 16-week Triglycerides (mg/dl) | 116.6±46.3 | 93.2±31.9 | 92.2±29.3 | 0.27 |
| 16-week Glucose (mg/dl) | 78.4±6.9 | 79.1±6.9 | 78.4±6.2 | 0.96 |
| 16-week Lactate (mmol/l) | 1.1±0.4 | 0.9±0.3 | 0.9±0.6 | 0.73 |
| OGTT (mg/dL) | 96.5±23.3 | 113.1±34.2 | 116.6±20.4 | 0.24 |
| Gestational weight gain (kg) | 15.4±4.3 | 22.7±19.9 | 21.5±14.8 | 0.48 |
| Gestational length (weeks) | 39.8±1.1 | 39.7±1.1 | 39.2±1.4 | 0.53 |
| Parity | 1 (0, 2) | 0 (0, 1) | 1 (0, 3) | 0.66 |
| Mode of delivery (SVD/C-section) | 2 (1, 2) | 2 (1, 2) | 2 (1, 2) | 0.93 |
| **Infant Characteristics** | **AE (10)** | **CE (9)** | **RE (11)** | **p-value** |
| Fetal sex (F/M) | 3/7 | 3/6 | 3/8 | 0.96 |
| Birth weight (kg) | 3.6±.4 | 3.5±.5 | 3.4±.4 | 0.56 |
| Birth length (m) | 0.49±0.03 | 0.49±0.01 | 0.50±0.03 | 0.59 |
| Birth BMI | 14.7±1.6 | 14.7±2.0 | 13.5±1.2 | 0.13 |
| Head circumference (m) | 0.34±0.01 | 0.35±0.02 | 0.34±0.01 | 0.52 |
| Chest circumference (m) | 0.33±0.02 | 0.33±0.02 | 0.33±0.01 | 0.9 |
| Abdominal circumference (m) | 0.31±0.02 | 0.31±0.02 | 0.31±0.01 | 0.98 |
| Apgar-1 minute | 8 (7, 9) | 9 (8, 9) | 8 (7, 9) | 0.79 |
| Apgar-5 minute | 9 (9, 9) | 9 (9, 9) | 9 (9, 9) | 0.99 |
| All data expressed as mean ± SD, t-test, p≤0.05. * VO2peak n=22/30 due to COVID; OGTT = 1-hour blood oral glucose tolerance test at 24-28 weeks; Gestational weight gain = delivery - pre-pregnancy; SVD = spontaneous vaginal delivery; F= female infant sex; M = male infant sex. | | | | |

**
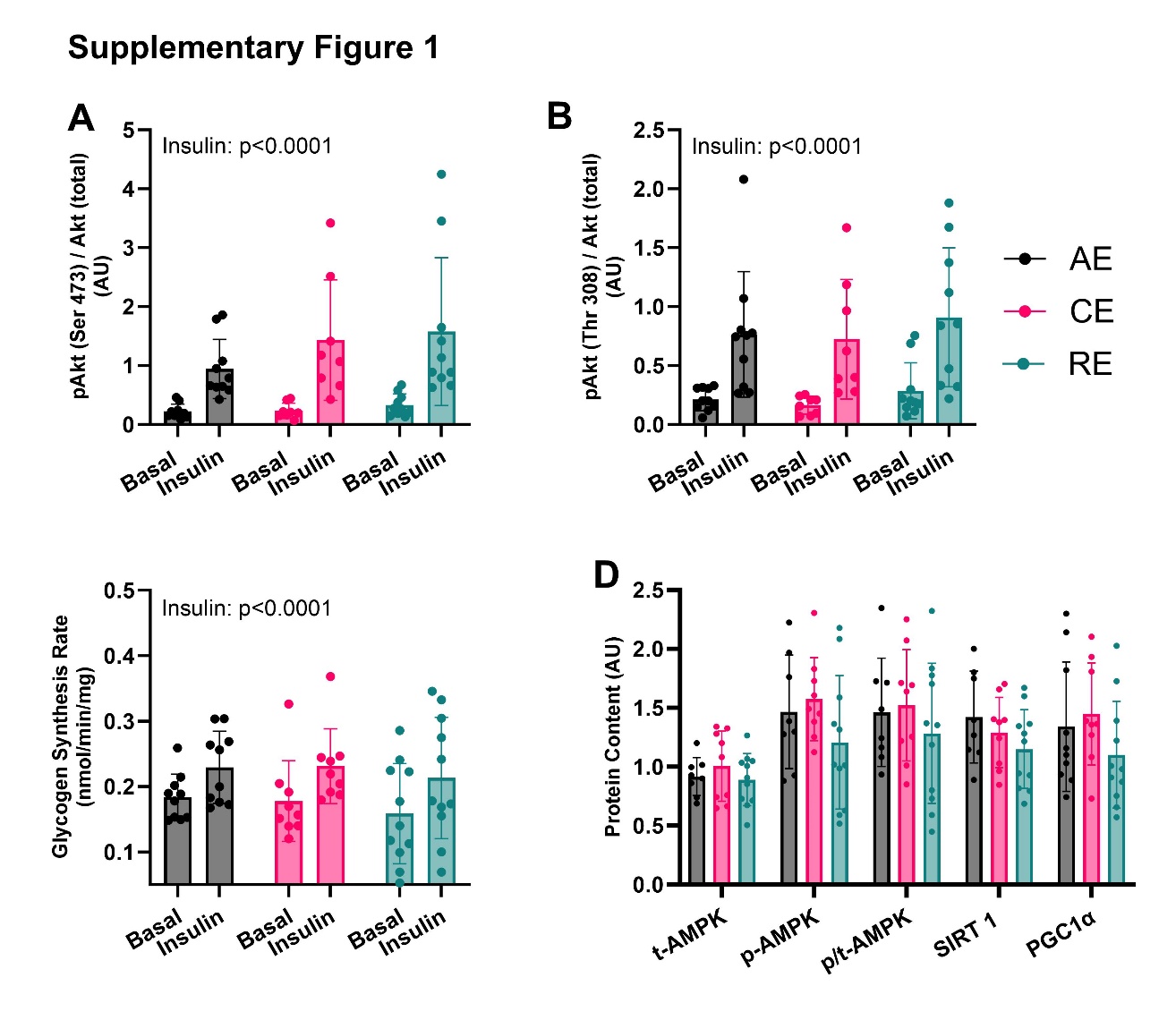
Supplementary Figure 1. Insulin action and expression of redox-sensitive and energy-sensing proteins.** Insulin-stimulated Akt phosphorylation at Ser473 (A) and Thr308 (B) was similar between exercise groups. Both absolute rates and relative increases in glycogen synthesis in response to insulin were similar across groups (C). Expression and activation of AMPK, SIRT1, and PGC1-α (D). Data expressed as mean ± SD. n=8-10/group. Two-way repeated measures ANCOVA (covariate – HDL at 16 weeks) and One-way ANCOVA. AE- aerobic; CE – combination; RE – resistance group.

**
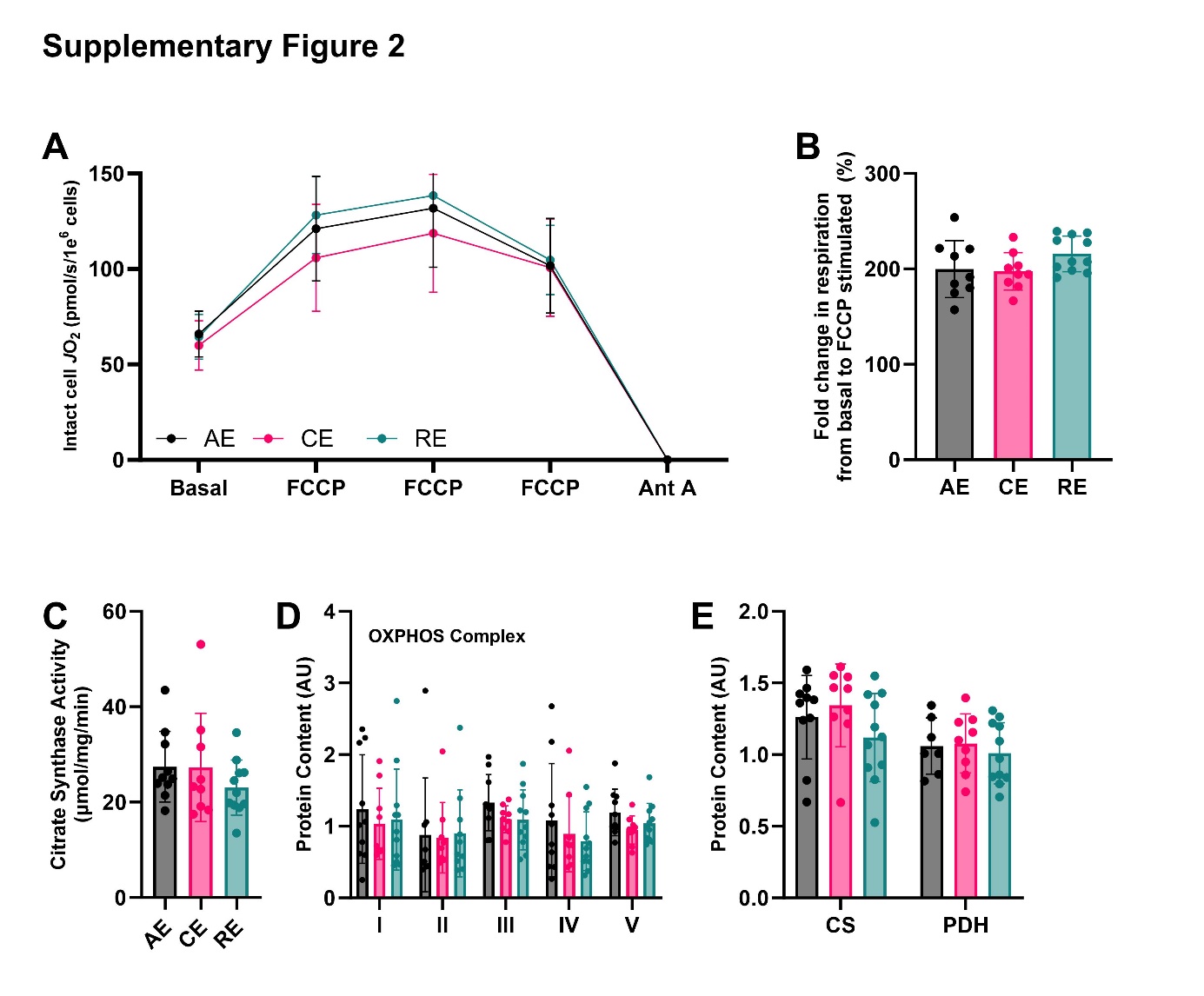
Supplementary Figure 2. Intact cell respiration and mitochondrial content.** Intact cell respiration (A) and response to FCCP (B) were similar between groups. Citrate synthase activity (C) was not different between groups. Protein expression of OXPHOS (D), citrate synthase (CS), and pyruvate dehydrogenase (PDH) (E) was similar between exercise groups. Data expressed as mean ± SD. n=8-10/group. ANCOVA (covariate – HDL at 16 weeks). AE- aerobic; CE – combination; RE – resistance group.

**
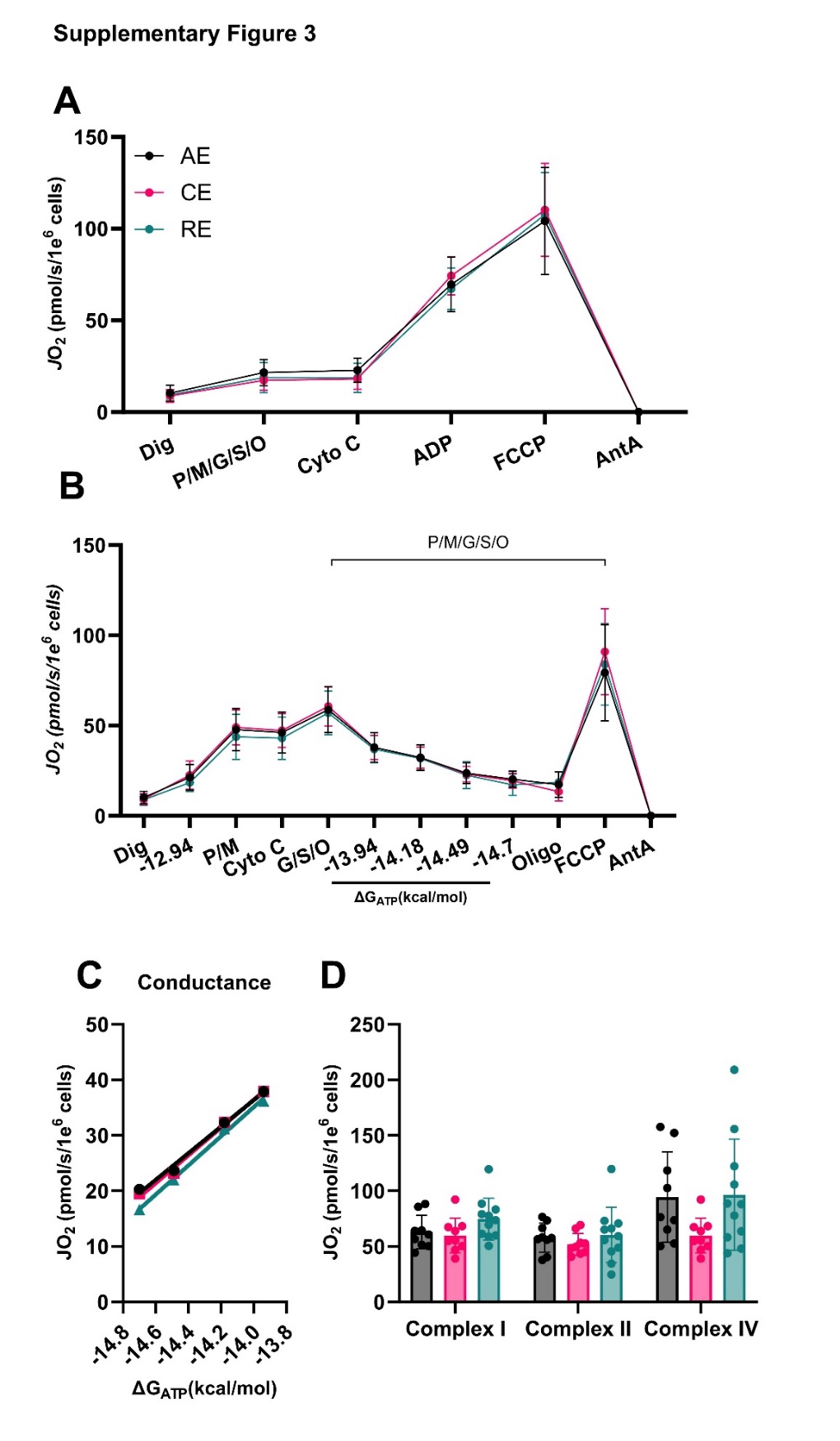
**

**Supplementary Figure 3. Permeabilized cell respiration.** ADP and FCCP-stimulated respiration in permeabilized cells were similar between exercise groups (A), regardless of the presence of ΔGATP (B). Conductance (C). Cellular respiration supported with complex I, II, or IV specific substrates (D). Data expressed as mean ± SD. n=8-10/group. ANCOVA (covariate – HDL at 16-weeks). AE- aerobic; CE – combination; RE – resistance group.

**
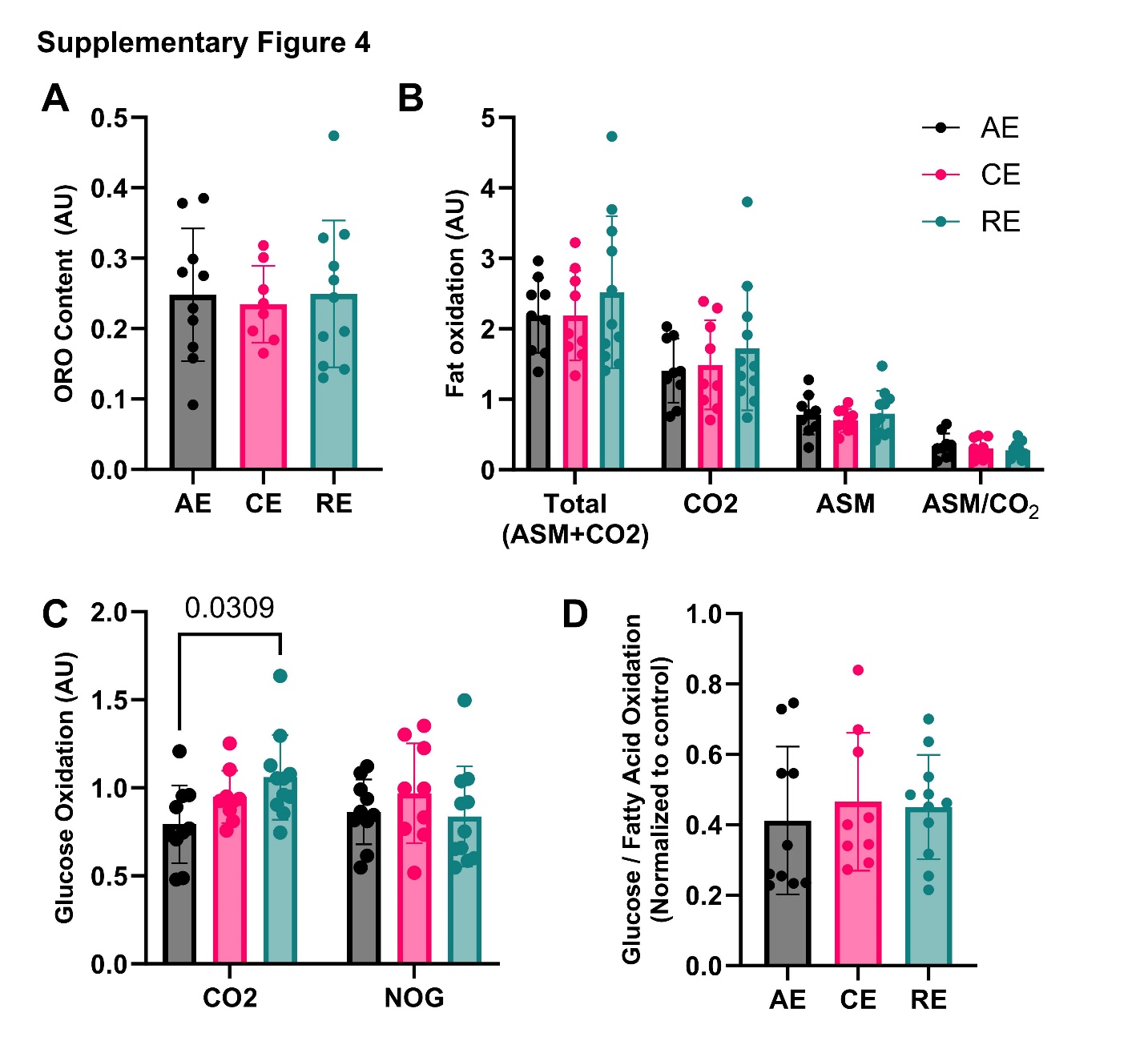
**

**Supplementary Figure 4. Fat and glucose oxidation, and fat storage.** Neutral lipid storage measured by oil-red-o stain was similar between exercise groups (A). Fatty acid uptake (total), complete oxidation (CO_2_), incomplete oxidation (ASM), and partitioning ratio (ASM/CO_2_) were similar between exercise groups (B). Glucose complete oxidation was higher in the resistance group compared to the aerobic group, without any difference in the rate of non-oxidized glycolysis (NOG) between groups (C). Despite higher complete oxidation in RE compared to AE, ratio of glucose/fatty acid oxidation was similar across groups (D). Data expressed as mean ± SD. n=8-10/group. ANCOVA (covariate – HDL at 16 weeks), with Bonferroni post-hoc test. AE- aerobic; CE – combination; RE – resistance group.

**
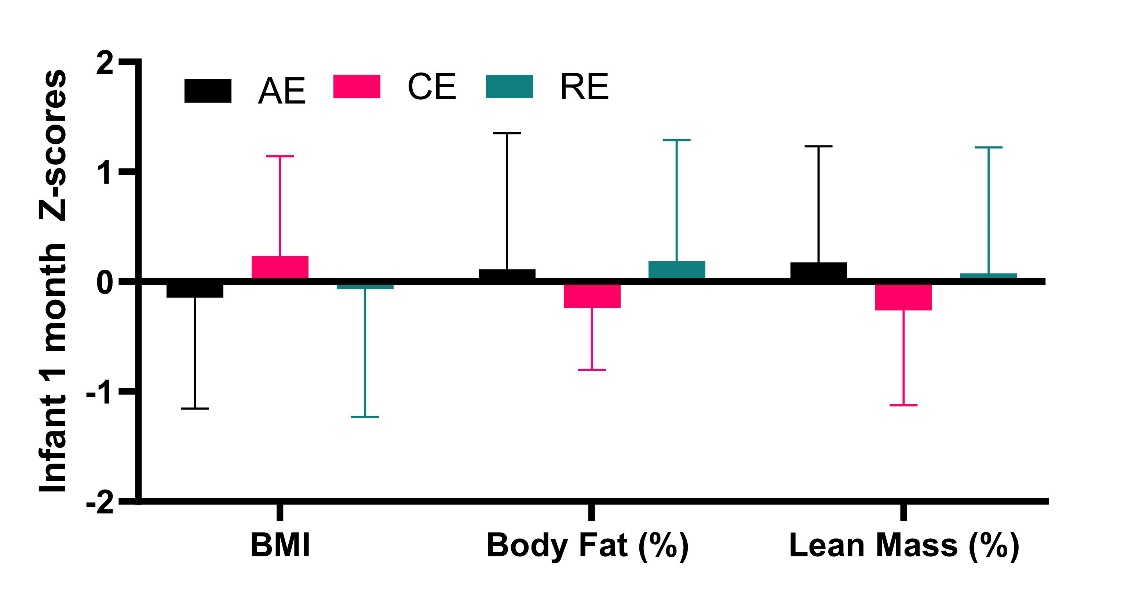
**

**Supplementary Figure 5. Infant body composition.** BMI, body fat percentage, and lean mass z scores were similar across exercise groups. Data expressed as mean ± SD. n=8-10/group. ANCOVA (covariate – HDL at 16 weeks). AE- aerobic; CE – combination; RE – resistance group.


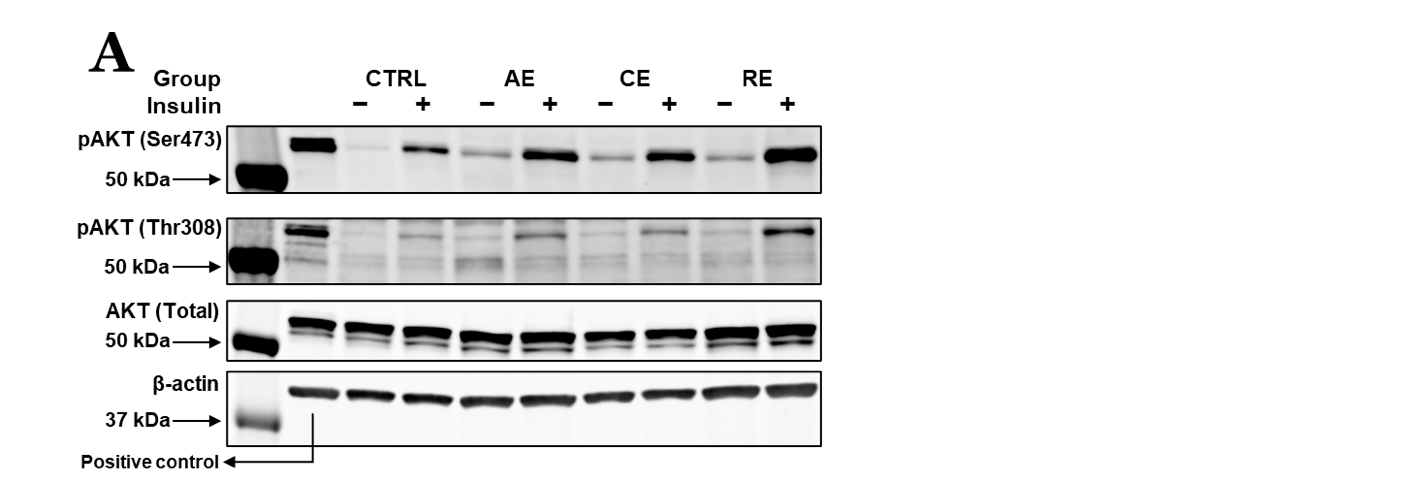
**Supplementary Figure 6 -Western blot panels.**


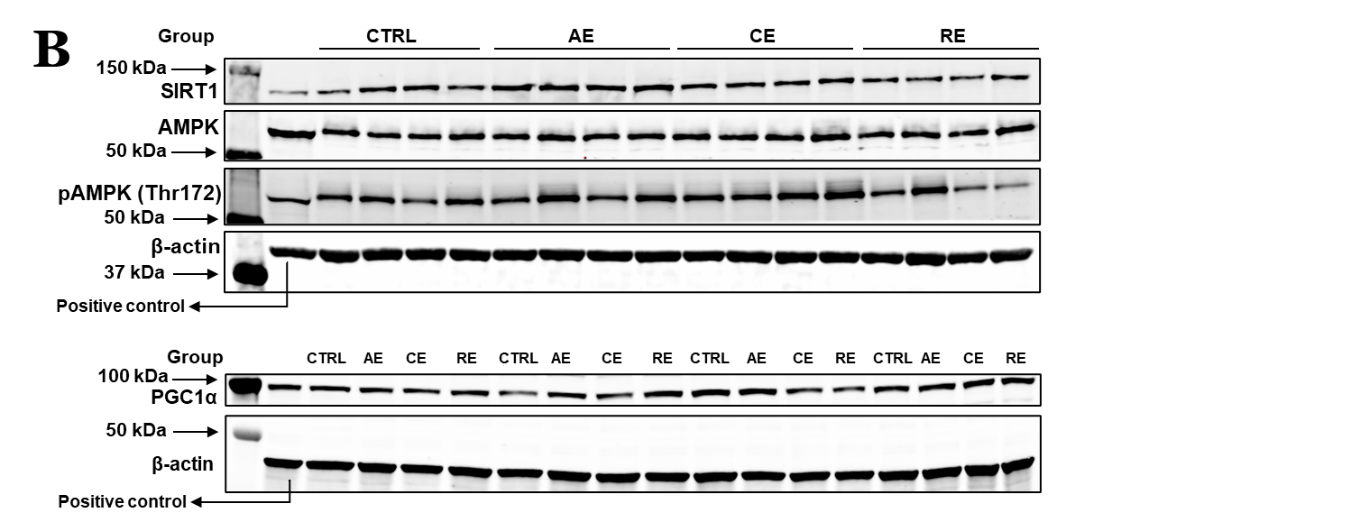


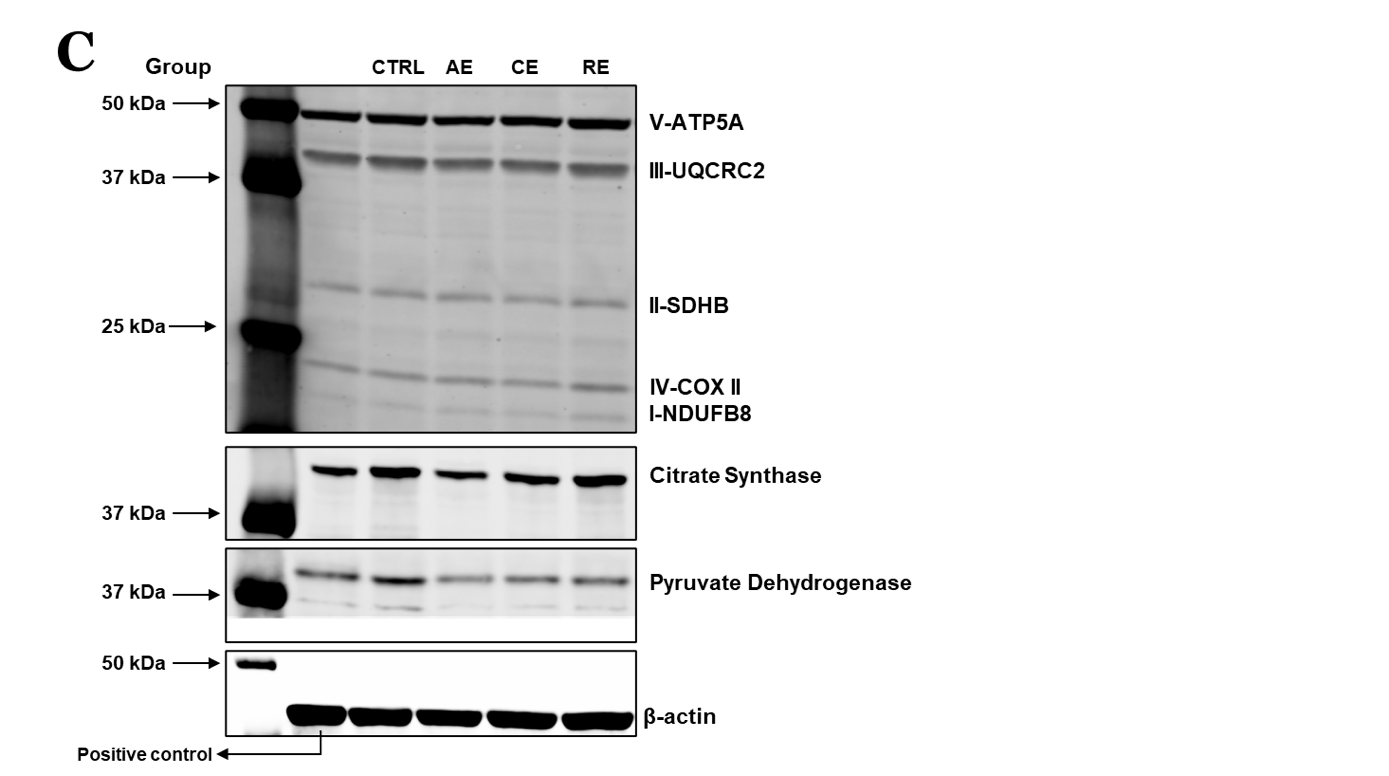


**
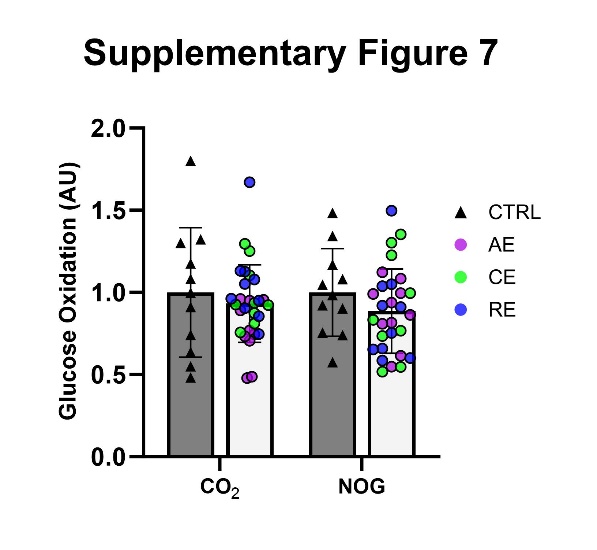
**

**Supplementary Figure 7.** Glucose oxidation to CO_2_ and the rate of non-oxidized glycolysis were similar between the control and combined exercise group (B). Data expressed as mean ± SD. control n=11, exercise, n=30; AE- aerobic; CE – combination; RE – resistance group.
